# Retroperitoneal paraduodenal unicentric Castleman disease: case report and review of the literature

**DOI:** 10.1093/jscr/rjae073

**Published:** 2024-02-16

**Authors:** Eva Intagliata, Rosario Vecchio, Clarissa Vizzini, Loredana Villari, Rossella Rosaria Cacciola, Emma Cacciola, Veronica Vecchio

**Affiliations:** Department of General Surgery and Medical Surgical Specialties, University of Catania, Policlinico “G. Rodolico—San Marco”, Via S. Sofia 78, 95123 Catania, Italy; Department of General Surgery and Medical Surgical Specialties, University of Catania, Policlinico “G. Rodolico—San Marco”, Via S. Sofia 78, 95123 Catania, Italy; Department of General Surgery and Medical Surgical Specialties, University of Catania, Policlinico “G. Rodolico—San Marco”, Via S. Sofia 78, 95123 Catania, Italy; Pathological Anatomy Unit, University of Catania, Italy Policlinico “G. Rodolico—San Marco”, Via S. Sofia 78, 95123 Catania, Italy; Department of Biomedical Science, Hematologic Unit, University of Catania, Policlinico “G. Rodolico—San Marco”, Via S. Sofia 78, 95123 Catania, Italy; Department of Medical Sciences, Surgical Sciences and Advanced Technologies, Hemostasis Unit, University of Catania, Italy, Policlinico “G. Rodolico—San Marco”, Via S. Sofia 78, 95123 Catania, Italy; Department of Biomedical Science, Hematologic Unit, University of Catania, Policlinico “G. Rodolico—San Marco”, Via S. Sofia 78, 95123 Catania, Italy

**Keywords:** unicentric Castleman disease, lymphadenopathy, laparoscopic excision

## Abstract

Castleman disease is a rare and benign disorder, characterized by enlarged lymph nodes and angiofollicular lymphoid hyperplasia. We report a case of a 57-year-old male, who was admitted to our surgical department because of a retroperitoneal nodular mass measuring about 4 cm in maximum diameter, incidentally discovered on a radiologic exam performed for the onset of vague abdominal pain with posterior irradiation. The patient was subdue to laparoscopic removal of the mass and no intra- and post-operative complications were recorded. Histologic diagnosis of hyaline-vascular variant of the Castleman disease was confirmed. Only two cases have been found in the literature reporting the paraduodenal unicentric Castleman disease localization like our case. Although rare, the Castleman disease must be considered in the differential diagnosis among all the lymph nodes diseases, for avoiding improper therapies.

## Introduction

Castleman disease is a rare and benign disorder, characterized by enlarged lymph nodes and angiofollicular lymphoid hyperplasia. It is a very rare disease and its incidence rate is estimated to be 21–25 per million person per year [1]. It was first reported by Benjamin Castleman in 1954 as a mediastinal lymphadenopathy that appeared to be thymomas, but it was neither thymic nor neoplastic in origin [2]. Castleman disease includes two main disorders having similar histological traits: unicentric Castleman disease (UCD) and multicentric Castleman disease [3]. Suspicion of this disease can be given by anamnestic, laboratory, and radiological evaluations, but for the diagnosis, an excised lymph node biopsy specimen exhibiting classic histological features is required [3, 4]. We present a rare case of retroperitoneal Castleman disease.

## Case report

**Figure 1:**
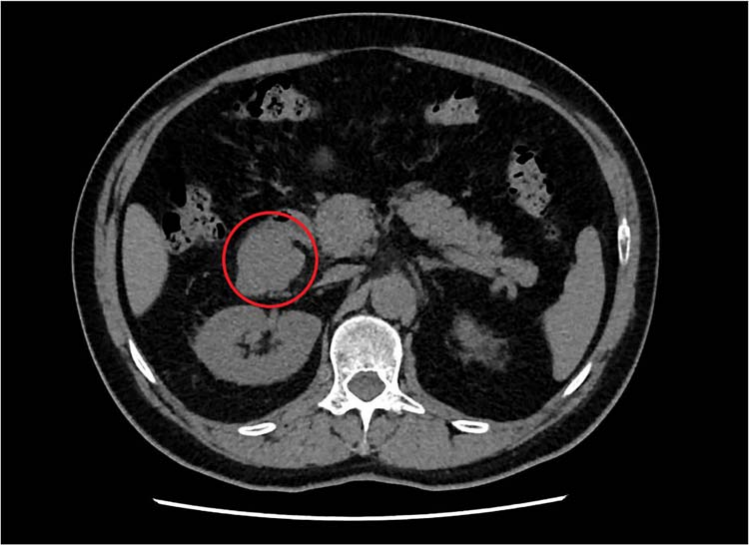
Transversal abdominal CT scan revealing a retroperitoneal polylobate neoformation in the right perirenal space.

**Figure 2:**
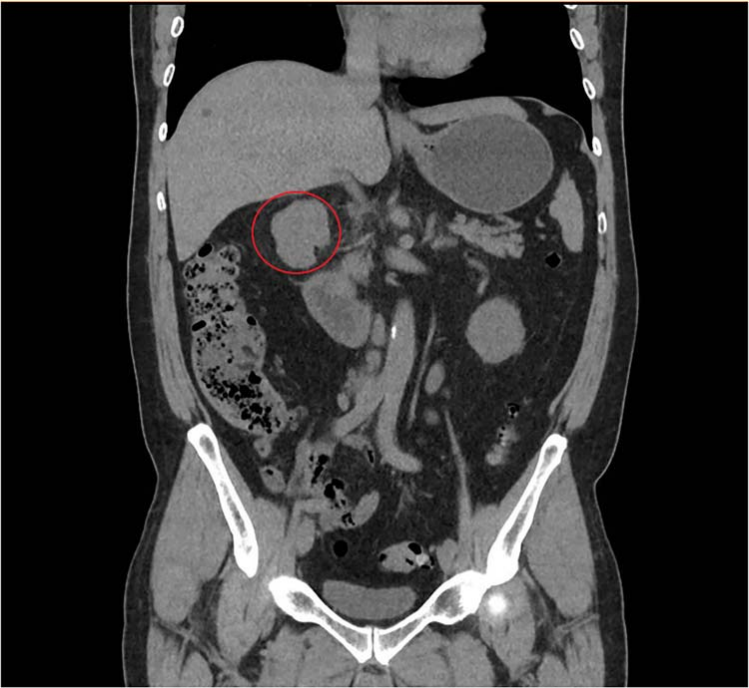
Coronal abdominal CT scan revealing a retroperitoneal polylobate neoformation near the duodenum.

A 57-year-old male, with only a history of severe arterial hypertension, was admitted to our surgical department because of a retroperitoneal nodular mass measuring about 4 cm in maximum diameter, discovered on an abdominal echography. The ultrasonography was performed for the onset of a 1-year long vague abdominal pain at the right hypochondrium with posterior irradiation, without any other associated symptoms. Subsequently, he underwent total body enhanced CT scan, which confirmed the presence of a retroperitoneal polylobate neoformation developing in the right perirenal space, with maximum axial diameters of about 40 × 70 mm, which exerted a mass effect on the duodenum with which a thin adipose cleavage plane was present (Figs 1 and 2). The mass was rich of contrast impregnation in the early phase, which further increased in the portal phase. The presence of a vascular structure was highlighted posteriorly to the mass.

**Figure 3:**
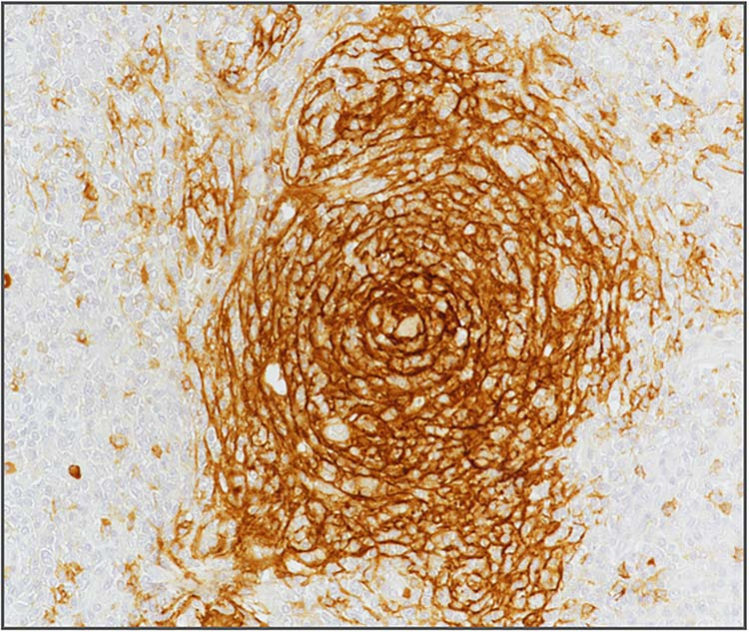
Hyperplasia of follicular dendritic cells (staining of follicular dendritic cells with anti-CD21 antibody).

**Figure 4:**
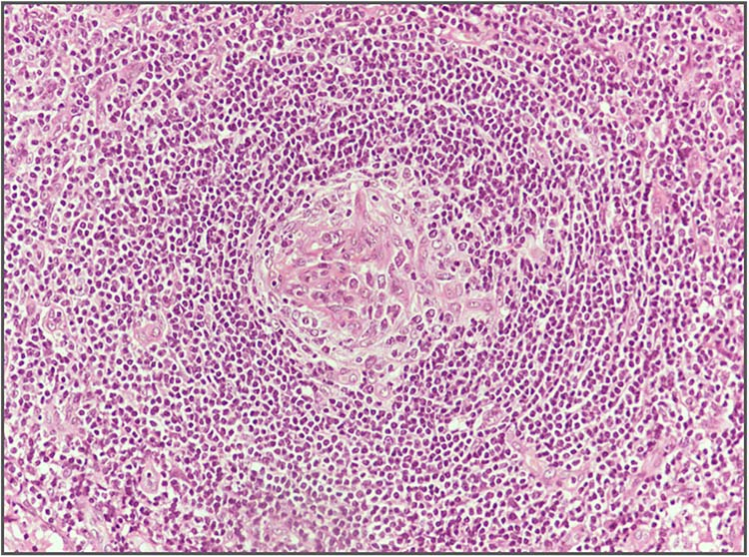
Dysplasia of follicular dendritic cells.

**Figure 5:**
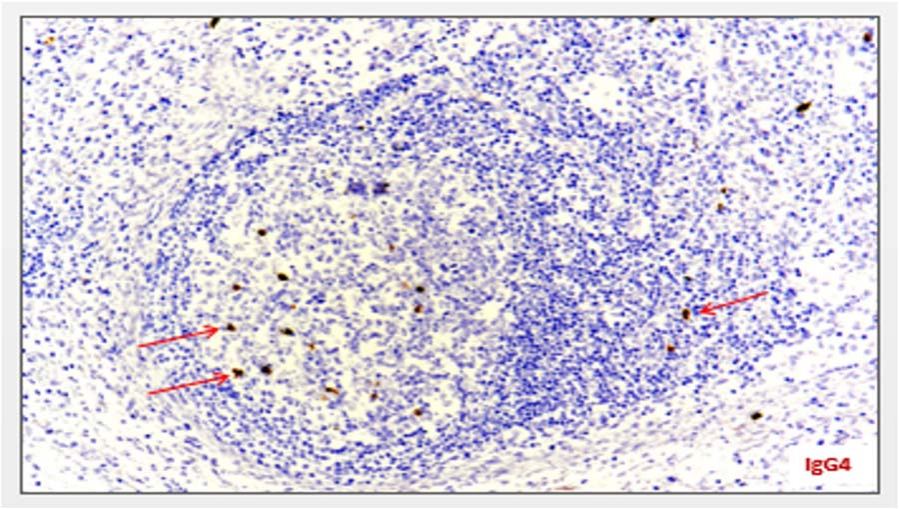
Minimum amount of IgG4-secreting plasma cells (staining with anti-IgG4 antibody) (arrows).

Blood investigations including hematological and biochemical tests revealed a low lymphocytes count with a consequent increased neutrophils percentage, and an increased protein C reactive (PCR) value. Oncological markers were normal. He was also tested for HIV infection, HHV-8, and Epstein–Barr virus with negative results. Positron emission tomography (PET) was performed with evidence of increased metabolic activity at the site of the mass. A suspicious diagnosis of Schwannoma or liposarcoma was made. The patient underwent CT-guided percutaneous biopsy with extemporary histological diagnosis of hyaline-vascular variant Castleman disease. The patient was subdue to laparoscopic removal of the mass. The mass was located near the duodenum without other structures involvement, from which it was isolated and entirely resected comprehending the intact capsule. No intra- and post-operative complications were recorded. The microscopic examination revealed the presence of a single lymph node measuring 7 × 5 × 4.5 cm with clear margins and an architecture characterized by the presence of small follicles distributed over the entire surface, lacking clear centers and polarization (primary follicle or atrophic follicle type), some with prominent vascularization and fibrosis (hyaline-vascular transformation), hyperplasia of the CD21+ follicular dendritic cells (Figs 3 and 4), and a “target” arrangement of the mantle lymphocytes. Large follicles with hyperplastic germinative centers and follicles with small centers duplicate germinatives, marked paracortical interfollicular vascular hyperplasia, and poor polytypic interfollicular plasmacytosis with very low proportion of IgG4+ plasma cells, coexist (Fig. 5).

Diagnosis of hyaline-vascular variant of the Castleman disease was confirmed. The patient was discharged on the 9th postoperative day.

## Discussion

Castleman disease is a rare lymphoproliferative disorder characterized by involvement of lymph nodes at any part of the body, usually with a benign behavior. Because of its heterogenous histopathological findings and number of localization, several classifications have been proposed, but the most used is the one that divides it into two forms: unicentric and idiopathic multicentric.

Benjamin Castleman described the disease in the 1950s as a localized mediastinal lymph node enlargement characterized by lymphoid follicles hyperplasia with germinal center involution and capillary proliferation [2].

Risk factors are unknown [3]. Data suggest that UCD is more likely a clonal neoplastic process [5], originating from the follicular dendritic cell [5–7].

There are no specific criteria listed to diagnose the UCD. The main common findings are a single lymph node involvement, pain, C-reactive protein elevation, and a single lesion detected at the CT-scan and CT-FDG-PET.

The definitive diagnosis of CD is histopathologic on an excised lymph node biopsy specimen [3]. The CD is a typically polyclonal lymphocyte proliferation having four histopathological subtypes: the hyaline vascular type is the most common form of UCD [2, 8]. Less commonly, plasmacytosis, follicular, and interfollicular changes, lacking of sinuses, dysplastic stromal/dendritic cells, follicles depleted of lymphoid cells, marked proliferation of vasculature within the interfollicular zones, hyperplasia of the follicular dendritic cells are found.

UCD presents as a slow-growing solitary mass that can be found at any lymph node station, such as the abdominal cavity [9–24], the chest [25–33], the limbs [3, 10], and the neck [34–37]. Unusual localization is possible, like the orbit region [38, 39]. Only two cases have been found in the literature reporting the paraduodenal UCD localization like our case [9, 15].

The prevalence of CD is not known, but it seems to be less than 1/100 000, with a preference for the female gender [3]. The average age of UCD diagnosis is younger (fourth decade) than for multicentric CD patients (sixth decade) [40–42]. In our systematic review, the age range was 12–66 years for the male patients, and 25–72 years for the female patients [10, 29].

UCD is most often asymptomatic and discovered incidentally during a physical examination or an imaging exam. In 30% of cases, aspecific systemic symptoms are observed (asthenia, febrile episodes, mild weight loss, night sweats). Pain or discomfort is the most frequent symptoms because of the compression of surrounding structures. Severe complications can occur like polyneuropathy, pulmonary complications, and autoimmune hemolytic anemia [43]. Vague persistent abdominal pain at the right hypochondrium irradiating posteriorly was the only symptom reported by our patient.

Paraneoplastic syndrome is possible and has been reported in several case reports [25, 29, 44–50].

Laboratory tests are usually normal, but anemia, hypergammaglobulinemia, and elevated sedimentation rate may be present [3]. Our patient presented a high C-reactive protein level and a low lymphocytes count.

Ultrasound exam, computed tomography, magnetic resonance, and positron emission tomography are useful for detection of pathological lymph nodes and for biopsy planning. Preoperative biopsy may not always be diagnostic, remaining a controversial issue.

Surgery is the first-line therapy and has a curative potential with symptoms remission and normalization of laboratory abnormalities. However, follow-up is recommended.

Because of its unicentric and localized non-invasive behavior, laparoscopic surgery may be the first choice as surgical therapy, because of its well-known benefits [51, 52].

The management of unresectable UCD is more challenging. Irradiation, embolization, or neoadjuvant therapy with anti-CD 20 (rituximab) or anti-interleukin 6 (siltuximab) or tocilizumab should be considered [3, 43, 53–55].

UCD patients appear to have a higher risk of developing follicular dendritic cell sarcomas and Hodgkin or non-Hodgkin lymphoma [3].

Although rare, the CD must be considered in the differential diagnosis among all the lymph nodes diseases, for avoiding improper therapies.
